# Recent advances in oncolytic virus therapy for hepatocellular carcinoma

**DOI:** 10.3389/fonc.2023.1172292

**Published:** 2023-04-26

**Authors:** Licheng Zhu, Yu Lei, Jia Huang, Yahang An, Yanqiao Ren, Lei Chen, Huangxuan Zhao, Chuansheng Zheng

**Affiliations:** ^1^ Department of Radiology, Union Hospital, Tongji Medical College, Huazhong University of Science and Technology, Wuhan, China; ^2^ Department of Interventional Radiology, Union Hospital, Tongji Medical College, Huazhong University of Science and Technology, Wuhan, China; ^3^ The First Affiliated Hospital, and College of Clinical Medicine of Henan University of Science and Technology, Luoyang, China

**Keywords:** oncolytic virus, oncolytic virotherapy, hepatocellular carcinoma, mechanism, combination therapy, clinical trial

## Abstract

Hepatocellular carcinoma (HCC) is a highly refractory cancer and the fourth leading cause of cancer-related mortality worldwide. Despite the development of a detailed treatment strategy for HCC, the survival rate remains unsatisfactory. Oncolytic virus has been extensively researched as a new cancer therapeutic agent in the treatment of HCC. Researchers have designed a variety of recombinant viruses based on natural oncolytic diseases, which can increase the targeting of oncolytic viruses to HCC and their survival in tumors, as well as kill tumor cells and inhibit the growth of HCC through a variety of mechanisms. The overall efficacy of oncolytic virus therapy is known to be influenced by anti-tumor immunity, toxic killing effect and inhibition of tumor angiogenesis, etc. Therefore, a comprehensive review of the multiple oncolytic mechanisms of oncolytic viruses in HCC has been conducted. So far, a large number of relevant clinical trials are under way or have been completed, and some encouraging results have been obtained. Studies have shown that oncolytic virus combined with other HCC therapies may be a feasible method, including local therapy, chemotherapy, molecular targeted therapy and immunotherapy. In addition, different delivery routes for oncolytic viruses have been studied so far. These studies make oncolytic virus a new and attractive drug for the treatment of HCC.

## 1 Introduction

The liver cancer is the fourth leading cause of cancer-related mortality worldwide, and the liver is the sixth most common site of primary cancer (1). Hepatocellular carcinoma (HCC) accounts for approximately 90% of all liver cancers (2). Standard therapies for HCC have made significant progress, but due to late symptom manifestations, most patients have reached an advanced stage upon initial diagnosis (3). This means that there is no chance for radical treatments such as transplantation, liver resection and radiofrequency ablation. Existing therapeutic methods, such as transarterial chemoembolization (TACE), radiotherapy, chemotherapy, and immunotherapy, have limited improvements in overall survival (OS) of advanced HCC patients owing to low response rate and high recurrence rate (4, 5). Therefore, there is an urgent need of satisfactory treatment strategies to improve therapeutic efficacy for HCC. HCC lesions have a variety of management options, which are influenced by multiple factors including the number, their size, the extrahepatic spread of the lesions, as well as the status of the patient’s physical condition and liver function (6).

Oncolytic viruses (OVs) are a new class of anticancer drugs that are well known for their ability to preferentially replicate and proliferate in tumor cells, induce immunogenic cell death, and stimulate of host anti-tumor immunity, thus promoting tumor regression (7, 8). Tumor therapy with OVs originated from a woman suffering from leukemia; after infection with influenza virus, tumor regression occurred, which led to researchers’ exploration of tumor therapy with OVs (9). In the past decades, OVs have been extensively studied in the treatment of tumors. Talimogene laherparepvec (T-VEC) was the first virus agent applied for clinical therapy and was approved for treating malignant melanoma in the western world in 2015 (10).

Oncolytic virotherapy (OVT) is a novel therapy in cancer treatment. Specifically, OVs selectively infect tumor cells by utilizing internal gene mutation or metabolic reprogramming of tumor cells, and then replicate in tumor cells to kill target cells, or indirectly kill tumors by stimulating the immune system’s anti-tumor response. Some viruses have an innate tropism for tumor cells, like reoviruses, Newcastle disease virus (NDV), and coxsackievirus (CV). However, due to the low tumor specificity and tumor lysis efficiency of most viruses, the application of natural OVs is limited. Armed-OVs combines the superiority of gene therapy and virotherapy and is likely to bring a new hope for tumor therapy (11). With the development of viral gene recombination technology, tumor-selective replicating OVs can be constructed by deleting or inserting various genes to meet researchers’ expectations for OVs. Common gene modified viruses include adenovirus (AdV), vesicular stomatitis virus (VSV), vaccinia virus (VV), etc. (12). Numerous preclinical studies have demonstrated that it is efficient and advisable to design OVs to express specific genes that promote cytotoxic killing of tumor cells, activate immune responses, inhibit tumor neoangiogenesis, and enhance sensitivity to radiotherapy (13–15). To date, there are three OV products are on the market, i.e. talimogene laherparepvec (T-VEC), Oncorine (H101) and Delytact (9, 16).

Great efforts also have been made to further explore new OV agents and the combination of OVs with other therapeutic strategies such as locoregional therapies, chemotherapy, targeted-drugs therapy, and immunotherapy for HCC. Meanwhile, with the delicate combination of surgical navigation technology with artificial intelligence technology (17), it may further push the potential to enhance the efficacy of OVs treatment. Nonetheless, the clinical value of these high-tech technologies combined with drugs (such as OVs) still need to be discussed in a large number of trials in the future.

In this review, we described the biological basis of OVs, the mechanism of tumor therapy, the progress of preclinical research, and the progress of clinical research on OVs for the treatment of HCC. In addition, in view of the current research status of the application of OVs in treating HCC, combined with the new progress in the current treatment, we looked forward to the possible development of OVs for the treatment of HCC in the future.

## 2 The biological basis of OVs in HCC

### 2.1 Specificity of OVs to HCC

The dysfunction of antiviral response may lead to OVs preferential replication in tumor cells. In tumor cells, certain signaling pathways are responsible for immortality, cellular growth, immune suppression, and metabolic dysregulation. In particular, dysfunctional signaling pathways may contribute to ineffective antiviral responses. Thus, viruses are more likely to infect tumor cells than normal cells (7). Several different methods can be used to design OVs to specifically target liver cancer cells (18).

The first strategy focuses on the tumor signaling pathway. Some key virus genes or some bases can be deleted or mutated, thus making it possible for virus to replicate in tumor cells. In this design, the E1A and E1B genes are primarily deleted. For example, ONYX-015 is a typical engineered adenovirus. Gene editing technology is used to delete the E1B gene so that the E1B protein is unable to form and the engineered virus can only replicate in p53-dificient cells such as HCC cells (19). However, according to further studies, the deletion of E1B protein mediated late viral mRNA nuclear export rather than the degradation of p53 (20).

The second method targets the expression of key genes related to virus replication (21–24). As Alpha-fetoprotein (AFP) and Golgi phosphoprotein 2 (GOLPH2) are expressed in a high level in HCC, two new oncolytic adenoviruses are developed by replacing the endogenous E1B promoter with the AFP promoter (ZD55) and the GOLPH2 promoter (GD55), respectively. As expected, these OVs showed higher specificity against HCC and an enhanced antitumor effect at the same time (21).

The third method is to engineer viral capsids so that OVs can specifically target the liver cancer cell receptor (25). Adenovirus can bind capsid fibers to specific receptors on the cell surface, thus efficiently targeting and infecting host cells; this suggests that the structure of adenovirus capsid directly influences the binding ability of adenovirus to HCC (26). Furthermore, researchers promoted the spread of the virus by modifying the capsid to induce the formation of syncytium between tumor cells; they replaced the membrane surface glycoprotein with hemagglutinin-neuraminidase (HN) protein and modified fusion membrane protein, so that the safety and ability of virus to spread between cells and kill cancer cells were enhanced (27).

Although some viruses show a congenital tendency to tumors, many viruses have limited selectivity to tumors, and therefore those cells must be modified by molecular engineering to better infect tumor cells rather than normal cells. So far, the research on how to improve the selectivity of OVs through modification is still limited. However, the development of local treatments may make the selectivity of the virus viable.

### 2.2 Intratumoral survival of OVs in HCC

After the OVs enters the cell, the cell undergoes a series of defensive reactions to protect itself. The secretion and release of type I IFN induce the anti-tumor response, which could activate multiple pathways to play an anti-virus response (28). The deficiency of type I IFN provides soil for OVs to infect and replicate in tumor cells (29). A recent study constructed a OVs expressing apocalypses vatus lectin, which up-regulated type I IFN expression in HCC cell lines, therefore enhancing anti-tumor efficacy without affecting virus replication. The reason for this is that it can also crosstalk the PI3K/Akt, MAPK/ERK and Hippo/MST pathways regulated by Raf-1, as well as the metabolic related pathways that promote viral replication (30).

Meanwhile, OVs can recruit immune cells and reverse the immunosuppressive microenvironment in tumors. At the same time, the elimination of virus by the immune system is also one of the important factors affecting the efficacy of OVs. OVs infection can lead to congenital and adaptive immune activation of the virus. The complex strategy aimed at natural killer (NK) cells is the most commonly used method to avoid the early elimination of viruses. Researchers in the field of liver cancer have developed two methods, one of which is using recombinant virus to express some proteins, such as matrix 3 (M3) and equine herpes virus-1 glycoprotein G. M3 is a chemokine-binding protein from murine gammaherpesvirus-68. With the help of M3, chemokine signaling can be antagonized and the accumulation of neutrophil and NK cell can be reduced in the lesions, allowing virus to survive and to exert oncolytic function (31). Equine herpes virus-1 glycoprotein G can reduce NK and NKT cells through extensive combination with viral chemokines, leading to increased viral titer in tumor (32). The second is to inhibit the anti-virus innate immunity of the body through the depletion of immune cells. The use of proteasome inhibitor bortezomib will damage the development of B cells, especially when it is combined with viruses (33). Both methods showed that the viral titer and the necrosis rate in the tumor were increased significantly, which stimulates the new concept that inhibition of host antiviral inflammatory response can conspicuously enhance the efficacy of tumor lysis universally applicable in the field of cancer OVT.

## 3 The oncolytic mechanisms of OVs in HCC

As a new cancer therapeutic agent, OVs have many advantages because they can inhibit tumor growth in many ways. These advantages include direct tumor lysis, activation of anti-tumor immunity, destruction of tumor vasculature, and induction of apoptosis. Although the molecular and cellular details of these processes in HCC are still not completely clear, the exploration of using OVs to treat HCC is proceeding steadily, and the opinion of reasonable combination therapy based on OVs is being clarified.

### 3.1 Directly killing virus infected tumor cells

By inserting promoters that are specific to cells or tissues to direct gene expression in tumor cells, OVs improve the attachment to target host tumor cell membranes and make use of the host resources to replicate and proliferate, prevent the cell from producing host products, and promote the production of viral products. Eventually, OVs lyse the host cell and release many subviruses, which are capable of reinfecting tumor cells surrounding them and repeat the process to achieve effective killing of tumors (34).

However, literature shows that OVs cannot infect every tumor cell *in vivo*. Rapid clearance from the vascular system, reduced virulence of the virus as well as innate and adaptive immunity against the virus are key factors that could compromise the efficacy of OVs. Fortunately, the tumor killing effect caused by bystander effect has also become a phenomenon that cannot be ignored in OVT. The bystander effect means that although OVs could kill infected tumor cells, uninfected cells may also die. In HCC, the combination of the M1 virus and the VCP inhibitor has observed an exciting bystander effect. The results showed that cytotoxicity was caused by cytokines secreted by HCC cells after M1 virus infection, such as tumor necrosis factor- α (TNF- α), IL-8, IL-1A (35, 36). The bystander effect has become a possible mechanism of cytotoxicity in the combination of OVs and molecular targeted therapy.

### 3.2 Activating anti-tumor immune response

**Figure 1:**
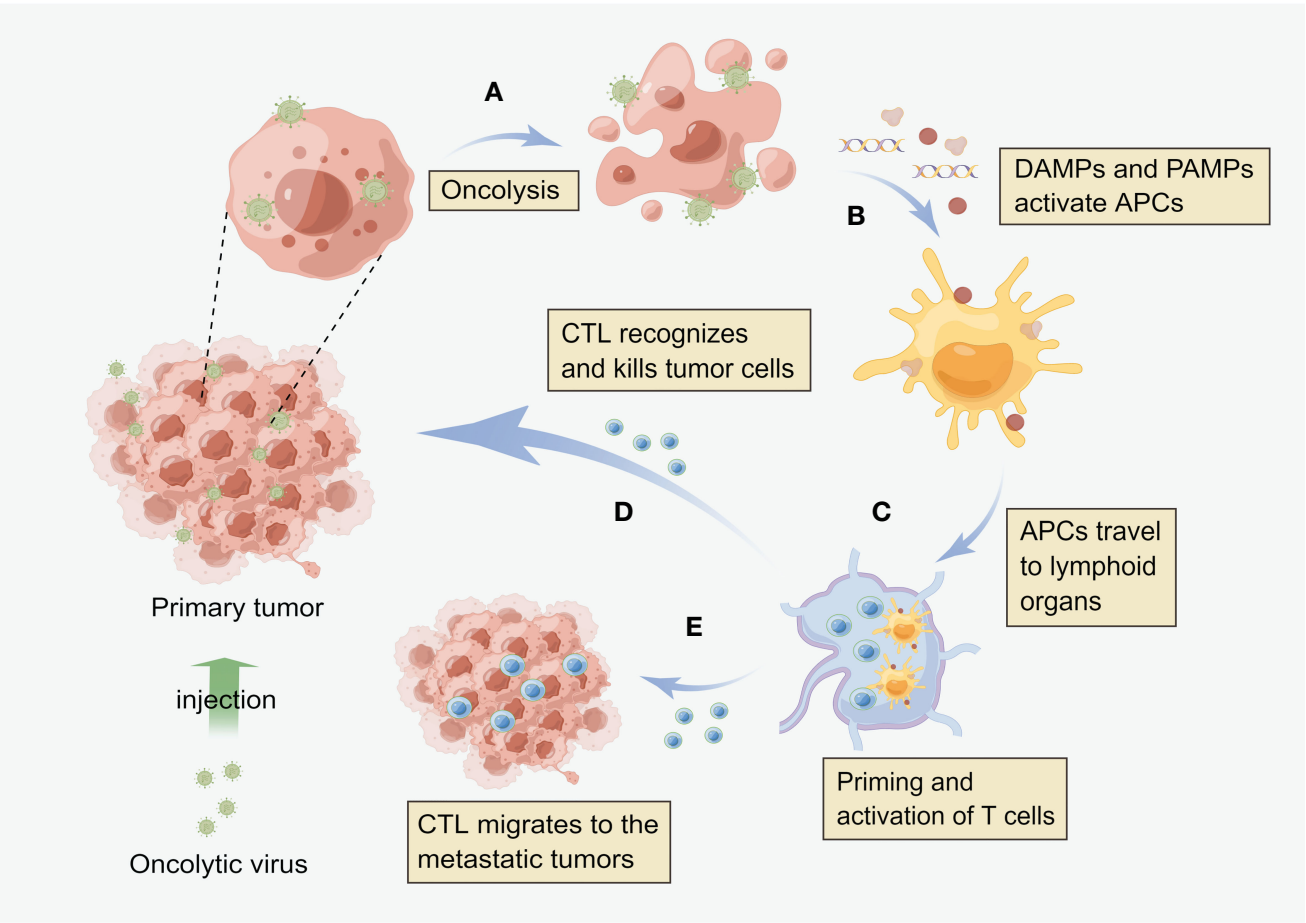
Oncolytic virus induces local and systemic anti-tumor immunity **(A)** After virus infects tumor cells, it replicates in a large number of cells and directly kills tumor cells. **(B)** After cell lysis, the release of soluble tumor associated antigen (TAA), viral pathogen associated molecular model (PAMP) and cell-derived injury associated molecular model (DAMP) enables antigen presenting cells (APCs) to be recruited to the viral infection site and activate. **(C)** APC engulfs antigens and migrates to lymph nodes, where adaptive T cell immunity against tumors is initiated. **(D)** These cytotoxic T cells (CTLs) can recognize and kill tumor cells. **(E)** Distant metastatic tumor is also the target of CTL, i.e. CTL can migrate to the distant tumor site.

HCC established a highly immunosuppressive tumor microenvironment that supports cancer cell growth. The tumor generates immunosuppressive molecules and recruits immunosuppressive cells to form an immune desert in the tumor tissue, paralyzing the anti-tumor immune response. It has been one of the immunotherapy directions that investigators are exploring nowadays to activate the body’s own immune system to kill tumors. As shown in **Figure 1**, OVs can effectively induce anti-tumor immune activity, because the local release of soluble tumor-associated antigens, cell-derived damage-associated molecular patterns (DAMPs) and viral pathogen-associated molecular patterns (PAMPs) caused by viral infection can be recognized and captured by antigen-presenting cells (APCs), thus promoting native immunity and adaptive immunity (37, 38). By producing inflammation in the tumor, OVs break the immune tolerance to tumor antigens, and even produce tumor specific memory responses (39). NDV is one of the first non-engineered anti-cancer virus therapies. Due to its non-pathogenic or mild toxicity in humans, it is still safer and more effective than any currently produced engineered OVs. A recent study has shown that NDV has better tumor cytotoxicity than cisplatin, and the activation of IFN-related pathway and innate immunity is an important way to inhibit tumor (40). In addition, a study has shown that reovirus can increase immune cell infiltration within tumors and induce the expression of immune checkpoint proteins through the IFN mechanism. The use of immune checkpoint blockers in the sequential treatment of OVs can improve the survival rate of tumor model mice. These results provide new strategies for the combined systemic immune virus therapy for primary and secondary tumors (41).

However, the immune response induced by OVs is usually insufficient and cannot lead to a sustained cure in clinical and preclinical studies. In order to enhance the effectiveness of anti-cancer immunity, oncolytic adenoviruses are often modified to improve the expression of immunostimulatory molecules (42). These cytokines have shown a significant ability to enhance the therapeutic effect of OVs, but the most successful one is GM-CSF. In particular, GM-CSF can resist tumor by influencing the type and level of immune response, without blocking virus replication (43, 44). It was found that the local expression of GM-CSF further enhanced the migration and maturation of dendritic cells, presented antigens to CD4+ and CD8+ T cells, and triggered systemic T cell response (45). Furthermore, studies have shown that increased neutrophil infiltration in tumors treated with GM-CSF virus would relieve the immune suppression by reducing reactive oxygen species (ROS) and arginase I(ARG1) in the tumor microenvironment (46, 47). In addition, there have been a lot of studies using OVs armed with genes for Th1-cytokines, such as IL-2, IL-12, IL-15, IL-21, and IL-36. These cytokines promote the Th1-Tc1 antitumor immunity and thus enhance therapeutic efficacy in the context of OVs (48, 49).

On the other hand, tumor immune escape is an important target of current treatment. As the most studied immune checkpoint inhibitors, anti-PDL1/PD1 and anti-CTLA4 represent the most promising new cancer therapies in the past two decades (50, 51). Based on the ability of viruses to transcribe and express proteins in local tumors, many recombinant viruses carrying PDL1 or CTLA4 antibodies have been designed. For example, Zhang et al. designed an adenovirus expressing fusion proteins. The M terminal of the fusion protein contains the extracellular domain of PD-1, which can couple with PDL1 on tumor cells and block signal transduction; the C-terminal contains the extracellular domain of CD137L, which is used to enhance T cell activity. In various HCC models, the recombinant virus inhibits tumor growth through CD8+T cells (52). The same results were also observed in the virus carrying CTLA4 antibody (rFlu-huCTLA4). RFlu-huCTLA4 can kill tumor cells in a dose dependent manner and activate systemic anti-tumor immunity (53).

### 3.3 Disrupting the vascular system in tumor

HCC is a highly vascular tumor with high expression of vascular endothelial growth factor (VEGF). Under hypoxic conditions, a large number of angiogenic factors in the tumor microenvironment stimulate the recruitment of blood vessels. However, the tortuous leakage of these vascular structures cannot alleviate hypoxia (54). Small molecule tyrosine kinase inhibitors (TKI) targeting the kinase pathway of VEGFR cells, such as sorafenib, have been widely used in clinical trials, and the progression free survival and overall survival of HCC patients have been improved (55).

Some viruses can not only target tumor cells, but also affect tumor related vascular endothelial cells. One study has shown that vaccinia virus can directly infect and replicate tumor related CD31 endothelial cells in tumor tissues, leading to vascular destruction and collapse, which has been demonstrated in multiple clinical trials (18, 56). The most studied OVs in HCC, JX-594, is selective to tumor related vascular system (56). JX-594 is one kind of vaccinia virus with two genes inserted into the TK gene region, of which one encodes hGM-CSF and another encodes lac-Z (57). JX-594 can induce antitumor immunity and inhibit tumor blood vessels by promoting the maturation of myeloid and dendritic cell (58). Liu et al. found that the administration of JX-594 induced the expression of antivascular cytokines, and was connected with tumor vascular shutdown (59). Possible mechanism is that viral replication may be activated by epidermal growth factor receptor (EGFR)/Ras pathway signaling, cellular TK levels, as well as resistance to type-I interferons (IFNs) of cancer cells (60).

Meanwhile, other studies have suggested that OVs can produce neutrophil clumps in the blood vessels through their effect on tumor environment stromal cells. These neutrophils initiate fibrin deposition and release coagulation factors, therefore disrupting the tumor vascular system in the end (61). In fact, the purpose of neutrophil recruitment is to neutralize and kill the virus, in which immune thrombosis is initiated (62). JX-594 can collect concentrated neutrophil within 24 hours, and the induced closure of blood vessels can last for 10 days before blood supply reconstruction (60, 63). Another example is that vesicular stomatitis virus can block angiogenesis, thus cutting off tumors’ oxygen and nutrient supply by triggering tumor microenvironment (TME) inflammation and causing the formation of local microthrombosis (64).

There are many advantages for OVs to attack tumor blood vessels. Destruction of tumor vascular system can starve tumor cells and prevent tumor metastasis. However, the highly hypoxic tumor microenvironment will lead to the emergence of drug resistance phenotype, as well as enhanced migration and invasion. The closure of tumor blood vessels will also lead to poor transportation of immune effector cells and inefficient delivery of therapeutic agents (including OVs). There is an urgent need to find a balance between these two aspects.

### 3.4 Induction of apoptosis and blocking cell cycle

Escaping apoptosis leading to unregulated cell proliferation is considered to be one of the signs of cell carcinogenesis, usually due to the genetic defects of apoptosis signaling pathway and tumor microenvironment that inhibit apoptosis. Apoptosis caused by extracellular stimulation is caused by the combination of cell surface death receptors (DRs) and specific ligands. The well-known DR ligand is TNF- α, FasL and TNF-related apoptosis-inducing ligand (TRAIL) (65).

Some natural OVs can kill HCC cells through apoptosis. M1 virus can selectively kill HCC lacking in zinc finger antiviral protein (ZAP), and promote endoplasmic reticulum (ER) stress leading to cell apoptosis (35). In addition to ER stress, VV can also induce apoptosis through autophagy and Wnt signaling pathways (66). In contrast, reovirus induces apoptosis through DR and mitochondrial mediated pathways (67). Moreover, aimed at DR ligand, some recombinant viruses that induce apoptosis and blocking cell cycle have been designed, among which TRAIL is the most studied. HCC displays a high resistance to TRAIL-mediated cell death. To increase sensitivity of HCC cells to TRAIL, Z. Pei et al. have constructed an oncolytic adenoviral vector (ZD55) and used this vector to deliver TRAIL genes (ZD55-TRAIL) into HCC cells. And they found combination treatment with ZD55-TRAIL led to rapid and potent apoptosis activation in tumor cells and caused complete tumor xenograft elimination in all treated animals, making it a useful therapeutic strategy for HCC (68). TRAIL can selectively induce apoptosis of tumor cells through caspase-8, while its impact on normal cells can be ignored (69). Another great quantity studies showed that the oncolytic adenovirus (Ad-ΔB/TRAIL) can not only increase the expression of caspase 8, but also trigger downstream caspase cascade reaction through caspase 9, leading to cell apoptosis, which is related to mitochondrial mediated apoptosis (70–72). In addition, OVs carrying TRAIL and cytokines, such as IL-12 and IL-24, have also been studied in HCC, and these genes makes the recombinant virus have an impact on tumor immunity while inducing cell apoptosis (14, 73).

However, compared with other types of cell death, cell apoptosis has immunogenicity inertia and cannot explain the highly inflammatory tumor microenvironment caused by OVs. Therefore, the immunogenic cancer cell death (ICD) induced by OVs is also worth discussing, such as immunogenic cell apoptosis, necrotic apoptosis, scorching and autophagic cell death (74). The correct way of death is the key to trigger immune response. OVs can not only induce programmed necrosis of tumor cells, but also activate anti-tumor immunity through Gasdermin family (GSDM) (75, 76). In addition, the combined use of OVs and iron death inducers resulted in the activation of dendritic cells and the infiltration of T cells in tumors, which produced the best therapeutic effect and long-term immune memory (77).

Proper regulation of cell cycle is very important for cell proliferation, differentiation and cell homeostasis. During the progress of cell cycle, the expression of various cyclins will change accordingly. Many viruses regulate the cell cycle to facilitate their own replication. Some cytokines can induce cell-cycle arrest in various cancer cell lines, based on which, some OVs targeting cell cycle checkpoints have been designed. OVs can be genetically engineered to express such cytokines, thus gaining the ability to block cell cycle. L. Deng et al. constructed a recombinant oncolytic vaccinia virus (VG9-IL-24) can efficiently infected HCC cell lines and resulted in a high level of IL-24 expression. It is noteworthy that p53, as a downstream activator of IL-24, mediates cell cycle block at G2/M phase (78–80). *In vivo*, significant tumor growth inhibition and prolonged survival were observed in VG9-IL-24-treated mice (81). Similarly, the adenovirus carrying the Vector Like Family Member 4 (VGLL4) gene constructed by W. Xie et al. can also induce G2/M phase arrest in HCC cells and enhance apoptosis (82). Suppressor of cytokine signaling (SOCS) 1 and SOCS3 could down-regulate Cyclin D1 and anti-apoptotic proteins to induce HCC cell cycle arrest (83, 84).

## 4 OVs-mediated cancer combined therapy

OVs demonstrated good efficacy as an independent treatment in preclinical studies. However, the efficacy of OVT in patients may be greatly reduced due to antiviral immunity, tumor cell selection, and various physical and chemical barriers. To overcome these obstacles, combining OVs with other cancer therapies may be a viable option. In fact, a large assortment of natural and recombinant OVs show that various mechanisms of killing tumors can complement existing tumor therapies to achieve better efficacy.

### 4.1 OVs combined with locotherapy

Intraarterial treatments, such as transarterial embolization (TAE) and transarterial chemoembolization (TACE) are widely used as standard palliative treatments for patients with nonresectable HCC (85, 86). Although it is well accepted that TAE and TACE increase tumor response after treatment, clinical trials have shown that they have limited effects on the overall survival (87). The presence of some residual tumor cells led to tumor progression shortly after TACE. The efficiency of TACE tends to decrease as the procedure repeat, which is called as “TACE-refractory”. Thus, many trials have been conducted to combine TAE or TACE with other therapeutic methods, of which OVs may serve as a promising option. TACE could send Ovs directly into tumor through the transmission of blood vessels and prevent Ovs being eliminating by host immune system, thereby increasing the available concentration and reducing its impact on the body (88, 89). J. Altomonte et al. found that Degradable starch microspheres (DSM), which is an embolic agent, enhanced tumor necrosis and synergistically prolonged survival compared with VSV or DSM monotherapy when administered in combination with VSV in rat (90). In another study, immunohistochemistry showed that DSM caused the virus to stay in the tumor blood vessel for a long time, and increased the contact between the virus and tumor cells, which causes the virus infection, expression of therapeutic genes and the killing effect increasing on tumor (91). A recent study from our group showed that transarterial virus embolization modified the density of immune cells and reduced blood metastasis to some extent, and more importantly, established anti-tumor immune memory (92). Retrospective studies showed that compared with TACE alone, treating patients who have unresectable HCC with transarterial injection of human type-5 adenovirus (H101) could prolong OS and progression free survival (PFS) by 1.2 and 0.8 months, respectively (89), and greater survival benefit was observed in patients with elevated AFP, absence of metastasis, single tumor, tumor with a larger size, and HBsAg positive (93).

For early HCC, radiofrequency ablation (RFA) is one of the most effective local treatments. RFA and surgical resection have similar outcomes for HCC tumors smaller than 3cm, however, the former is more cost-effective (94). RFA can kill tumors by destroying tumor vascular systems and tumor cell membranes, inhibiting tumor growth and proliferation by suppressing the activity of key enzymes, and at the same time promoting endogenous antigen release, which increases the immunogenicity of HCC (95). J. Song et al. investigated delivering RFA immediately after OVs being intratumorally infused into HCC tumor region. And their study showed that the combination therapy of oncolytic virotherapy and RFA can effectively decrease the survival of HCC cells for both *in vitro* and *in vivo* experiments (52). In addition, a case report also shows that RFA combined with OVs has achieved good clinical results (96).

So far, only a few studies have reported the safety and efficacy of OVs when combined with local treatment. The mechanism of combination therapy inhibiting and killing HCC is unclear, which may be the research directions in the future. In addition, the malignant degree of residual tumors after local treatment will increase, regardless of TACE or RFA (97, 98). Whether OVs have the same killing effect on residual tumors or not remains to be explored, which may be one of the hotspots in the future.

### 4.2 OVs combined with chemotherapy

Chemotherapy is not the systemic treatment of HCC recommended in the guidelines, and the efficacy of cytotoxic chemotherapy is limited (99). The expression of drug resistance genes (such as heat shock protein, p53 mutation, etc.) (100) and the inability to tolerate due to liver dysfunction are the reasons for the short survival of HCC patients receiving chemotherapy (101). The potential of OVs may be fulfilled through their combinations with chemotherapeutic drugs, which may help to overcome some obstacles limiting the efficacy of OVs in the tumor microenvironment.

Compared with other chemotherapy, the use of cisplatin (DDP) seems to show more benefits (100). Up to now, there have been some reports on the efficacy and mechanism of combination of DDP and recombinant OVs in the treatment of HCC. The therapeutic genes expressed by recombinant OVs include IL-24, apoptosis inducing light (TRAIL), the second mitochondria derived activator of caspases (Smac) protein and X-linked IAP associated factor 1 (XAF1). There is a lack of consensus on the mechanisms of how they kill tumors. IL-24 induces apoptosis through activation of caspase family. The anti-tumor effect is further improved when IL-24 combine with DDP (102). DDP increases the sensitivity of TRAIL mediated cell death in HCC and produces a strong synergistic cytotoxicity in tumor cell lines (103). The expression of therapeutic genes Smac and XAF1 reduced the drug resistance of tumor cells to DDP and inhibited the growth of tumor (104, 105). It is gratifying that all combinations of OVs and DDP do not show superimposed toxicity to normal cells. In addition, the use of OVs helps to reduce the dose of DDP, which is beneficial to reduce side effects and make patients tolerate chemotherapy.

5-fluorouracil (5-FU) is also a chemotherapeutic drug that has been investigated in combination with OVs. Under the subtumor killing dose, 5-FU can increase the expression of p53. *In vivo*, the enhancement of p53 function has anti vascular activity. More importantly, 5-FU enhances virus replication and release. However, this study also found that the tumor will eventually recur, and no virus can be detected in the recurrent tumor, which may be due to immune elimination (106). Therefore, it is essential to explore how to make the virus survive in the tumor for a long time. Interestingly, researchers have also enabled OVs to express a specific enzyme, thus making an inert prodrug cytotoxic. Specific cytotoxic deaminase could be expressed by designing OVs, and these deaminase can turn the nontoxic prodrug 5-fluorocytosine to the anti-tumor drug 5-FU and therefore kill tumor cells (107). Using OVs to express specific enzymes and local injection can transform non-toxic precursor drugs administered systemically into high concentration active anticancer drugs in tumors, which may provide ideas for the research and combined treatment of OVs.

### 4.3 OVs combined with molecular-targeted therapy

Sorafenib was approved for advanced HCC systemic therapy in the European Union and the USA in 2007, and remained the only systemic agent for use for many years (108). In recent years, some new molecular targeted agents have been gradually used in clinical trials, such as apatinib and regorafenib, has been approved as a second-line treatment after sorafenib (109, 110). However, only a few patients yield a real and long-term benefit from this therapy. Similar to chemotherapy, the high resistance rate and some intolerable side effects have significantly limited the benefit of targeted therapy (111). Recently, it has been shown that the combination of OVs and targeted agents may serve as a new choice. In the past decade, the combination of sorafenib and OVs has been carried out in many studies, showing an exciting anti-cancer response. In the murine HCC tumor model, the use of sorafenib after injection of JX-594 can enhance the anti-tumor response (112). More importantly, combined therapy can significantly reduce tumor perfusion and induce tumor necrosis in 3 HCC patients (112). A III phase clinical trial of JX-594 combined with sorafenib in the treatment of refractory advanced HCC patients have been initiated but terminated early, as it did not improve efficacy outcome over sorafenib monotherapy in patients with advanced HCC. JX-594 can replicate in the tumor vascular endothelial cells, and destroy the tumor vascular system, but has no effect on normal blood vessels (56, 112). However, JX-594 replication tends to be inhibited by sorafenib if given simultaneously *in vitro*, therefore this combination may not have been optimal (112). Further studies are needed to address this issue in the future.

Similarly, Li L et al. indicated that the combination of H101, a recombined human adenovirus 5 type, and sorafenib exerted a superior function of inhibiting the proliferation of cancer cell and inducing apoptosis than sorafenib alone. This two drugs exert a synergistic effect in a specific range of sorafenib concentrations (113).

In addition, studies on the overcome drug resistance of sorafenib has also been conducted. J. W. Ady et al. examined the ability of GLV-1h68, which is a recombinant vaccinia virus used to eradicate smallpox, to kill HCC cell lines that are sorafenib-resistant (SR). No difference between the rates of cell death between the parental and SR cell lines was observed (114). This study may indicate that patients remain viable candidates for oncolytic virotherapy although they have failed treatment with sorafenib or have been resistant to sorafenib, making it a novel therapeutic method for advanced or recurrent HCC. However, another clinical study of JX-594, in patients with advanced HCC who had failed sorafenib found that JX-594 did not improve overall survival (115). In general, more preclinical and clinical studies are needed to test the applicability of OVs in advanced HCC patients with sorafenib resistance.

### 4.4 OVs combined with immunotherapy

Immunotherapy for HCC has become a popular treatment option in recent years. For HCC, chronic inflammation, immunosuppressive environment and T cell failure are important mechanisms leading to tumor occurrence and growth (116, 117). In some clinical trials, the new method of manipulating the immune response of HCC through immune checkpoint inhibitors (ICIs) has shown good efficacy (118–120). However, we have not retrieved any preclinical studies on the combination of OVs and ICIs in HCC so far. This may be because many recombinant viruses used in HCC can express immune checkpoint antibody after infecting cells. Nevertheless, this method cannot replace the combination of OVs and ICIs.

In addition, arming OVs with bi- or tri-specific T cell engager (BiTE or TriTE) is also a frontier strategy. BiTE is a recombinant bispecific protein. One end can target the surface molecules on T cells, and the other end can target the surface antigens of tumor cells (121). TriTE is similar to BiTE, but there is an additional site at one end that combines with molecules on the surface of T cells (121). Previous studies have shown that OVs armed with either BiTE or TriTE can not only kill infected tumor cells, but also induce bystander effect to kill uninfected tumor cells, and mediate superior anti-tumor activity (122, 123).

Adoptive cell transfer immunotherapy is another hotspot of cancer treatment, which includes adoptive T cells, chimeric antigen receptor T (CAR-T) cell and cytokine induced killer (CIK) cells (124–126). One study reported the therapeutic effect of measles virus combined with CD8NKG2D cells (a killer cell subset with NK and T cell phenotype and function) adoptive transfer on HCC, and studied the related mechanism. The findings indicated that measles virus could enhance the activation and infiltration of CD8NKG2D cells. Meanwhile, inhibition of indolamine 2,3-dioxygenase 1 (IDO1) by fludarabine could significantly improve the anti-tumor activity of adoptive cells (127). Furthermore, there have been a large number of preclinical and clinical studies on combining CAR-T cells and OVs to treat solid tumors in recent years. To our surprise, CAR-T cell has demonstrated remarkable efficacy in malignant tumors of the blood system, but it is not satisfactory in solid tumors (128). Studies on the combination of CAR-T and OVs have proved that OVs can help CAR-T cells accumulate and survive in solid tumors (129, 130). Compared with each single drug therapy, the combination of CAR-T cells and OVs has a better curative effect on tumors and prolongs the survival period. In addition, combination therapy also has a certain effect on tumor metastasis (130). Unfortunately, this therapy has not been studied in HCC, which may become a research direction in the future.

## 5 Delivering OVs to the tumor

**Figure 2:**
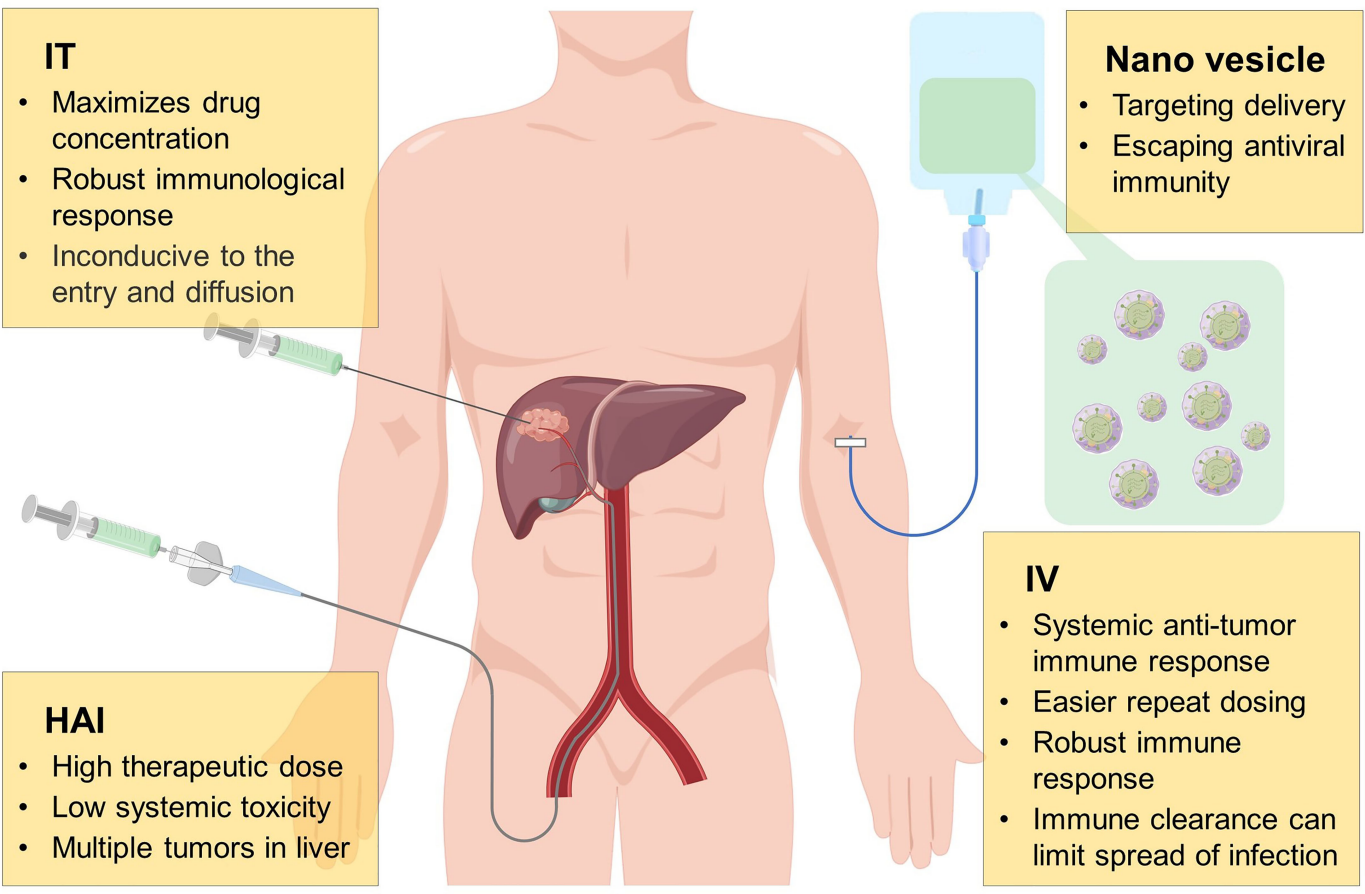
Mode of administration of oncolytic virus. During the development of oncolytic virus, many drug delivery routes have been investigated, including hepatic arterial infusion (HAI), intratumoral (IT), intravenous (IV) injection, of which each method has its own advantages and disadvantages. Anti-viral immune escape and tumor targeting by wrapping oncolytic virus with nano vesicles may be a more potential delivery method in the future.

So far, several drug delivery routes of OVs have been studied in HCC, as shown in **Figure 2**; common administration of OVs includes hepatic arterial infusion (HAI), intratumoral (IT), and intravenous (IV) injection. HAI is a conventional method of TACE and TAE, which can inject high doses of therapeutic agents into tumors to kill them while maintaining the systemic toxicity at a low level. However, it has been reported that the HAI administration of OVs leads to lethal systemic inflammatory reaction, which causes people to worry about its safety (131). Nowadays, some preclinical and clinical studies on HAI have reported that it is more effective than other administration methods. In terms of safety, injection of high dose of OVs through HAI will cause temporary changes in liver function, but it does not cause serious acute or chronic toxicity (132–135). IT injection is also a valuable drug delivery method. Similar to HAI, IT injection has the advantage of maximizing the distribution of OVs in tumors, but it also has certain limitations. For example, the dense and high-pressure environment of tumors may not be conducive to the entry and dispersion of OVs. In addition, as an internal organ, the liver is not convenient for OVs using IT injection. In this respect, IV injection seems to be a better method, and its high convenience and feasibility are conducive to the wide use of OVs in clinical. Furthermore, IV administration cause OVs arrive systemic metastasis and primary tumor, which enhances the induced anti-tumor response (136). However, due to the poor distribution of targets and the role of neutralizing antibodies in the circulation, OVs are rapidly cleared, and the efficacy of IV administration is still not satisfactory (137, 138). Hence, it is essential to develop new systemic and targeted delivery methods for solid tumors and metastatic malignancies.

There have been numerous studies aiming at improving the delivery efficiency of OVs to tumor tissues, which can be roughly classified into the following three categories: 1) Studies prevent OVs from being eliminated by the immune system by binding to specifically modifying key capsid proteins, which reduces viral immunogenicity and increases blood circulation time, but this approach also resulted in a significant reduction in viral infectivity (139); 2) Studies employ cell membrane-based OVs systemic delivery like extracellular vehicles (EVs) and tumor membrane-based hybrid vector systems (139); 3) Studies specifically deliver OVs into tumor cells by tumor microparticles (140).

Recently, there remain some innovative studies on the use of extracellular vesicles to deliver OVs in HCC. Extracellular vesicles are lipid membrane vesicles of nanometer to micrometer size, which can transport molecules from one cell to another for a long distance *in vivo (* 139). Massive small molecules such as curcumin, paclitaxel and macromolecules like DNA, RNA, protein have been proved to be successfully transported through extracellular vesicles (141–143). Lv et al. has produced a new type of bioengineered cell membrane nano vesicles (BCMNs), which have targeted ligands on the surface and can be effectively delivered to the tumor site. OVs can be directly encapsulated into BCMNs to escape pre-existing antiviral immunity (144). These results show that there are survivors’ benefit for BCMN-OVs, but there is no obvious toxicity to normal cells, which provides a clinical basis for improving OVTs. Other related studies include the construction of tumor-targeted bioreductive polymers by coupling polyethylene glycol with hepatoma-targeted peptides. The results show that the bioreductive polymer can safely and effectively deliver OVs to HCC (145, 146).

## 6 Clinical trials of OVs for HCC

At the time of writing this review (November 2022), we searched clinicaltrials.org for all clinical trials related to OVs and liver cancer. As shown in **Table 1**, there are 13 OVs in liver cancer patients for clinical trials. Unfortunately, only a few clinical trials have reported results. The clinical trial results of adenovirus dl1520 showed that HCC patients did not benefit from treatment, although they showed tolerance (147). JX-594 showed anti-tumor, anti-vascular, immunotherapeutic effect and good tolerance in HCC patients. In a phase I clinical study in 2005, researchers determined that 1 × 10^9^pfu is the maximum tolerable dose of IT injection for patients with primary or metastatic liver cancer (148). This study also showed that JX-594 can induce anti-vascular cytokines and suppressed HBV infection (148). A phase II clinical trial showed that the median total survival time of patients was significantly related to the virus dose, and the OS in the high-dose group was 7.4 months longer than that in the low-dose group, which is due mainly to the reason that JX-594 induces humoral and cellular anti-tumor immunity. Besides, the reduction and necrosis of tumor blood vessels caused by OVs is also an important reason for the survival of patients (149).

**Table 1:** Clinical trials of OVs for HCC since the 21^st^ century.

	Oncolytic Virus(transgeen)	Trial number	Status	Combination	Disease	Trail phase	Time	Mode of therapy
Adenovirus								
1	rAd-TK (TK99UN)	NCT00844623	Completed	alone	HCC	I	2004	IT
2	rAd5-p53 (Ad5CMV-p53 gene)	NCT00003147	Terminated	alone	Liver Cancer	I	2004	IT
3	rAd-p53 (p53 gene)	NCT02509169	Unknown status	alone	Advanced HCC	II	2015	HAI
4	rAd-p53 (p53 gene)	NCT02561546	Unknown status	alone	HCC, diabetes	II	2015	HAI
5	ADV-TK (thymidine kinase gene)	NCT00300521	Completed	LT	Liver Cancer	II	2006	IT
6	rAd-p53 (p53 gene)	NCT02418988	Unknown status	alone	HCC	II	2015	HAI
7	H101 (E1B)	NCT01869088	Unknown status	TACE	HCC	III	2013	HAI
8	H101 (E1B)	NCT03780049	Recruiting	HAIC of FOLFOX	Unresectable HCC at BCLC A-B Stage	III	2018	HAI
9	H101 (E1B)	NCT03790059	Unknown status	RFA	HCC	Not Applicable	2018	IT
10	ADV-TK (thymidine kinase gene)	NCT03313596	Unknown status	LT	Liver Cancer	III	2017	IT
11	H101 (E1B)	NCT05113290	Active, not recruiting	Sorafenib	Advanced HCC	IV	2021	IT
Herpes simplex virus								
13	T-VEC (GM-CSF gene)	NCT02509507	Active, not recruiting	Pembrolizumab	HCC et al.	I/II	2015	IT
14	GEN2 (HSV-Thymidine Kinase-m2 and hGM-CSF Genes)	NCT04313868	Recruiting	alone	HCC	I	2020	IT/HAI/IV
15	VG161 (Human IL12/15-PDL1B gene)	NCT05223816	Not yet recruiting	alone	HCC Intrahepatic Cholangiocarcinoma	II	2022	IT
Vaccinia virus								
16	JX-594	NCT00629759 NCT00554372	Completed	alone	HCC	I/II	2008 2007	IT
17	p53MVA vaccine (p53 gene)	NCT02432963	Active, not recruiting	Pembrolizumab	HCC et al.	I	2015	SC
18	JX-594	NCT01387555 NCT01636284	Completed	alone	Sorafenib Refractory HCC	II	2011 2012	IV
19	JX-594	NCT01171651 NCT02562755	Completed	Sorafenib	HCC	II/III	2010 2015	IT
20	PF-07263689	NCT05061537	Recruiting	Sasanlimab	Advanced HCC et al.	I	2021	IV
Vesicular stomatitis virus								
21	VSV-IFN-beta (IFN-beta gene)	NCT01628640	Active, not recruiting	alone	Sorafenib Refractory HCC	I	2012	IT
M1 virus								
22	M1-c6v1 (VTR106)	NCT04665362	Not yet recruiting	Apatinib, anti PD-1 antibody	Advanced HCC	I	2020	IV

TK, thymidine kinase; HCC, hepatocellular carcinoma; AFP, alpha-fetoprotein; GM-CSF, Granulocyte-macrophage Colony Stimulating Factor; TAE, transcatheter arterial embolization; LT, Liver transplantation; TACE, transcatheter arterial chemoembolization; HAIC, Hepatic arterial infusion chemotherapy; T-VEC, talimogene laherparepvec; IL, interleukin; PDL1, programmed cell death-ligand 1; IFN, Interferon; BCLC, Barcelona Clinic Liver Cancer; IT, intratumoral, IV, intravenous, HAI, hepatic arterial infusion; SC, subcutaneously.

From the perspective of development trend, OVTs has changed from natural virus to the application of recombinant virus, as well as from single therapy to combined therapy. Phase II/III clinical studies using adenovirus thymidine kinase (ADV-TK) gene therapy after liver transplantation (NCT00300521) showed that the overall three-year survival rate of the combined treatment group was 54.8%, higher than that of the liver transplantation only group. In the non-vascular invasion subgroup receiving combined therapy, the total survival and relapse free survival were 100%, significantly higher than those in the vascular invasion subgroup receiving combined therapy. Thus, it can be easily concluded that vascular invasion is an important factor affecting survival and recurrence. The study of JX-594 combined with sorafenib has been described in 4.3. We look forward to more relevant large-scale clinical trials, the results of which may lead to high impact multimodal treatment for HCC patients. The clinical trial data will further increase people’s understanding of OVT, and may have positive guiding significance for subsequent clinical trials, therefore improving OVT. Such approaches will delineate a new blueprint for HCC treatment.

## 7 Challenges and future perspectives

For over a century, viruses have been used for cancer treatment. With the development of genetic engineering and the understanding of the mechanism of virus action, OVs have gradually become an ideal cancer treatment agent. Accumulating studies show that OVs can not only directly dissolve tumor cells, but also involve a variety of complex regulatory mechanisms mentioned above. However, most preclinical and clinical studies show that single therapy, whether it is recombinant virus or natural virus, has limited efficacy, whereas OVs combined with other therapies may achieve better efficacy.

There are still some issues to be resolved. First and foremost, safety is at the top of the priority list. With the application of virus, the safety of OVT cannot be guaranteed (150). Besides, because the preparation of OVs is relatively difficult and complicated, there are problems in the extraction of a pure nontoxic single virus.

Secondly, it remains a challenge to adopt a systemic route of administration. In systemic administration of OVs, it is urgent to find an optional method to protect OVs from the destruction of host immunity and delivery OVs to targeted cancer cells. Furthermore, it is still a key issue to keep a balance between the anti-tumor immunity and antiviral immunity, which remains to be addressed in the future.

Thirdly, it is important for OVs to selectively target and lyse tumor cells while avoiding infecting normal cells. For the above-mentioned methods of specifically targeting tumor cells, further clinical trials are needed to confirm their validity.

Furthermore, other factors concerning the host, such as diet and the gut microbiome may also influence the therapeutic effect of OVs (37). In different patients, the same OVs agent may show different efficacy, which needs to be explored in future studies.

Despite the aforementioned potential issues, the potential of OVT in tumor treatment is promising. Recently, with the epidemic of the COVID-19, some reports have shown that some patients with malignant tumor surprisingly remitted after being infected with COVID-19. Despite that it is not yet clear whether the recovery of cancer can be attributed to COVID-19 infection or not, these findings have aroused great interest to OVs (9). Nevertheless, more extensive basic research and clinical trials are required to fulfill the immeasurable application potential and market of OVT as a treatment method and pave the way for clinical application. In the future, many issues remain to be elucidated, including virus species, the modification of gene, transgenic expression, route of administration, stage of disease, predictive biomarkers of response, and satisfactory combination strategies.

## Author contributions

CZ: Conceptualization, Methodology, Software. HZ: Data curation, Writing- Original draft preparation. LC: Visualization, Investigation. YA, YR: Revising; JH: Supervision. YL: Software, Validation. LZ: Writing- Reviewing and Editing. All authors contributed to the article and approved the submitted version.
